# Supplementary material for: Dietary Factors and Modulation of Bacteria Strains of Akkermansia muciniphila and Faecalibacterium prausnitzii: A Systematic Review
Source: Nutrients. 2019 Jul 11;11(7):1565. doi: 10.3390/nu11071565 (PMC6683038; doi:10.3390/nu11071565)
Supplement: Supplementary file 1 [file nutrients-11-01565-s001.pdf]

Table S1. PRISMA 2009 checklist

Section/topic	#	Checklist item	Reported on page #
**TITLE**			
Title	1	Identify the report as a systematic review, meta-analysis, or both.	1
**ABSTRACT**			
Structured summary	2	Provide a structured summary including, as applicable: background; objectives; data sources; study eligibility criteria, participants, and interventions; study appraisal and synthesis methods; results; limitations; conclusions and implications of key findings; systematic review registration number.	2
**INTRODUCTION**			
Rationale	3	Describe the rationale for the review in the context of what is already known.	4
Objectives	4	Provide an explicit statement of questions being addressed with reference to participants, interventions, comparisons, outcomes, and study design (PICOS).	4
**METHODS**			
Protocol and registration	5	Indicate if a review protocol exists, if and where it can be accessed (e.g., Web address), and, if available, provide registration information including registration number.	NA
Eligibility criteria	6	Specify study characteristics (e.g., PICOS, length of follow-up) and report characteristics (e.g., years considered, language, publication status) used as criteria for eligibility, giving rationale.	4-5
Information sources	7	Describe all information sources (e.g., databases with dates of coverage, contact with study authors to identify additional studies) in the search and date last searched.	4
Search	8	Present full electronic search strategy for at least one database, including any limits used, such that it could be repeated.	Appendix A
Study selection	9	State the process for selecting studies (i.e., screening, eligibility, included in systematic review, and, if applicable, included in the meta-analysis).	5

Data collection process	10	Describe method of data extraction from reports (e.g., piloted forms, independently, in duplicate) and any processes for obtaining and confirming data from investigators.	5
Data items	11	List and define all variables for which data were sought (e.g., PICOS, funding sources) and any assumptions and simplifications made.	5
Risk of bias in individual studies	12	Describe methods used for assessing risk of bias of individual studies (including specification of whether this was done at the study or outcome level), and how this information is to be used in any data synthesis.	5-6
Section/topic	#	Checklist item	Reported on page #
Summary measures	13	State the principal summary measures (e.g., risk ratio, difference in means).	NA
Synthesis of results	14	Describe the methods of handling data and combining results of studies, if done, including measures of consistency (e.g., I2) for each meta-analysis.	NA
Risk of bias across studies	15	Specify any assessment of risk of bias that may affect the cumulative evidence (e.g., publication bias, selective reporting within studies). 7	5-6
Additional analyses	16	Describe methods of additional analyses (e.g., sensitivity or subgroup analyses, meta-regression), if done, indicating which were pre-specified.	NA
**RESULTS**			
Study selection	17	Give numbers of studies screened, assessed for eligibility, and included in the review, with reasons for exclusions at each stage, ideally with a flow diagram.	6
Study characteristics	18	For each study, present characteristics for which data were extracted (e.g., study size, PICOS, follow-up period) and provide the citations.	7
Risk of bias within studies	19	Present data on risk of bias of each study and, if available, any outcome level assessment (see item 12).	7
Results of individual studies	20	For all outcomes considered (benefits or harms), present, for each study: (a) simple summary data for each intervention group (b) effect estimates and confidence intervals, ideally with a forest plot.	7-11

Synthesis of results	21	Present results of each meta-analysis done, including confidence intervals and measures of consistency.	NA
Risk of bias across studies	22	Present results of any assessment of risk of bias across studies (see Item 15).	7
Additional analysis	23	Give results of additional analyses, if done (e.g., sensitivity or subgroup analyses, meta-regression [see Item 16]).	NA
**DISCUSSION**			
Summary of evidence	24	Summarize the main findings including the strength of evidence for each main outcome; consider their relevance to key groups (e.g., healthcare providers, users, and policy makers).	12
Limitations	25	Discuss limitations at study and outcome level (e.g., risk of bias), and at review-level (e.g., incomplete retrieval of identified research, reporting bias).	17
Conclusions	26	Provide a general interpretation of the results in the context of other evidence, and implications for future research.	12-17
**FUNDING**			
Funding	27	Describe sources of funding for the systematic review and other support (e.g., supply of data); role of funders for the systematic review.	18

Table S2. MOOSE checklist

**Criteria**		**Brief description of how the criteria were handled in the meta-analysis**
**Reporting of background should include**		
√	Problem definition	Despite the emerging evidence evaluating the impact of dietary factors on changing microbiome flora, a comprehensive assessment of the dietary interventions in modulating *A. muciniphila* and *F. prausnitzii* is lacking.
√	Hypothesis statement	None.
√	Description of study outcomes	Abundance of *A. muciniphila* and *F. prausnitzii.*
√	Type of exposure or intervention used	Any dietary intervention.
√	Type of study designs used	We included intervention studies.
√	Study population	Adult individuals from the general population, with or without disease.
**Reporting of search strategy should include**		
√	Qualifications of searchers	The credentials of the investigators are indicated in the authors list.
√	Search strategy, including time period included in the synthesis and keywords	Search strategy and time periods are detailed in page 4 of the manuscript and in Appendix A.
√	Databases and registries searched	Embase.com and PubMed.
√	Search software used, name and version, including special features	We did not employ any search software. EndNote was used to merge retrieved citations and eliminate duplications.
√	Use of hand searching	We hand-searched bibliographies of retrieved papers and relevant reviews for additional references.
√	List of citations located and those excluded, including justifications	Details of the literature search process are outlined in the flow chart. Citations for the included studies are enclosed in the table 1 and 2. The citation list for excluded studies is available upon request.

√	Method of addressing articles published in languages other than English	We placed no restrictions on language. All identified studies were in English.
√	Method of handling abstracts and unpublished studies	No unpublished studies were identified
√	Description of any contact with authors	None
**Reporting of methods should include**		
√	Description of relevance or appropriateness of studies assembled for assessing the hypothesis to be tested	Detailed inclusion and exclusion criteria are described in the Methods section.
√	Rationale for the selection and coding of data	Data extracted from each of the studies were relevant to the population characteristics, study design, exposure and outcome.
√	Assessment of confounding	NA, this review included clinical trials.
√	Assessment of study quality, including blinding of quality assessors; stratification or regression on possible predictors of study results	Study quality was assessed using the Cochrane risk of bias tool.
√	Assessment of heterogeneity	We did not perform any test of heterogeneity.
√	Description of statistical methods in sufficient detail to be replicated	We did not perform statistical analyses.
√	Provision of appropriate tables and graphics	We included 2 main tables.
**Reporting of results should include**		
√	Graph summarizing individual study estimates and overall estimate	NA
√	Table giving descriptive information for each study included	Table 1 and Table 2.

√	Results of sensitivity testing	NA
√	Indication of statistical uncertainty of findings	NA.
**Reporting of discussion should include**		
√	Quantitative assessment of bias	We did not perform a quantitative assessment of bias.
√	Justification for exclusion	We excluded studies that used different exposure or outcome assessment for the comparison groups.
√	Assessment of quality of included studies	Study quality was assessed using the Cochrane risk of bias tool.
**Reporting of conclusions should include**		
√	Consideration of alternative explanations for observed results	We discussed that multiple testing might be a potential alternative explanation for the observed results, as not all studies corrected for this.
√	Generalization of the conclusions	The generalizability of our findings has been limited by the small sample sizes of the included trials. Also, the findings are not generalizable to children as the included trials only comprised the adult population.
√	Guidelines for future research	Future studies are required to untangle these complex interactions of diet, microbiome, and diseases in larger populations.
√	Disclosure of funding source	No separate funding was necessary for the undertaking of this systematic review.

Table S3. The Cochrane Collaboration's tool for assessing risk of bias

Author name, year	Sequence generation	Allocation concealment	Blinding of participants and personnel	Blinding of assessment	Missing data	Selective reporting	Other bias	Overall quality score
Benjamin JL et al. 2011								Fair
Benus RFJ et al. 2010							//////////////	Fair
Blatchford P et al. 2017				//////////////	//////////////			Poor
Clavel T et al. 2005				//////////////				Fair
Dao MC et al. 2016	NA	NA	NA	NA	//////////////			Poor
Dewulf EM et al. 2013				//////////////				Poor
Fava F et al. 2013			//////////////	//////////////				Poor
Fernando WMU et al. 2010			//////////////	//////////////			//////////////	Poor
Guadamuro L et al. 2015	NA	NA	NA	NA			//////////////	Poor
Halmos EP et al. 2015								Good
Halmos EP et al. 2016							//////////////	Fair
Hooda S et al. 2012				//////////////				Fair
Hustoft TN et al. 2016				//////////////			//////////////	Poor
James SL et al. 2015				//////////////				Fair
Lee T et al. 2017			//////////////	//////////////			//////////////	Poor

Li Z et al. 2015	NA	NA	NA	NA			//////////////	Poor
Majid HA et al. 2014							//////////////	Fair
Medina-Vera I et al. 2019				//////////////			//////////////	Poor
Moreno-Indias I et al. 2016			//////////////	//////////////				Poor
Most J et al. 2017				//////////////	//////////////		//////////////	Poor
Pinheiro I et al. 2017					//////////////			Poor
Ramirez-Farias C et al. 2009			//////////////	//////////////				Poor
Ramnani P et al. 2010				//////////////	//////////////			Poor
Roshanravan N et al. 2017					//////////////			Fair
Tagliabue A et al. 2017								Good
Vulevic J et al. 2013				//////////////				Fair
Walker JM et al. 2019					//////////////			Poor
West NP et al. 2013							//////////////	Fair
Wijayabahu AT et al. 2019	NA	NA	NA	NA	//////////////		//////////////	Poor
Xu J et al. 2015				//////////////	//////////////		//////////////	Poor

***other bias refers to bias due to problems not covered elsewhere in the table** (e.g. the study had a potential source of bias related to the specific study design used; or there is insufficient information to assess whether an important risk of bias exists; or insufficient rationale or evidence that an identified problem will introduce bias).
White: low risk of bias; pattern **///////**: unclear risk of bias; black: high risk of bias.
